# Absorption and Resonance Rayleigh Scattering Spectra of Ag(I) and Erythrosin System and Their Analytical Application in Food Safety

**DOI:** 10.3389/fnut.2022.900215

**Published:** 2022-05-09

**Authors:** Jian Wang, Shaopu Liu, Wei Shen

**Affiliations:** ^1^School of Elementary Education, Chongqing Normal University, Chongqing, China; ^2^School of Chemistry and Chemical Engineering, Southwest University, Chongqing, China

**Keywords:** Ag(I), erythrosin, absorption, resonance Rayleigh scattering (RRS), actual samples

## Abstract

In pH 4.4∼4.6 weakly acidic media, erythrosine (Ery) can react with Ag(I) to form hydrophobic ion-association complex, which can further aggregate to form nanoparticles with an average particle size of about 45 nm under the action of water phase extrusion and van der Waals force. As a result, it could lead to a decrease of absorbance, a significant enhancement of resonance Rayleigh scattering (RRS) and the appearance of a new emission spectrum. Based on these Phenomena, two new methods (spectrophotometry and RRS) were established for the determination of trace Ag(I). The detection limits for Ag(I) by spectrophotometry and RRS are 9.74 and 0.12 ng/ml, respectively. In this paper, we have investigated the formation of nanoparticles, the optimum reaction conditions, the influence factors, explored the reason for enhancement of the scattering intensity and the effect of coexisting substance. This research shows that RRS method not only has good selectivity and high sensitivity, but also is simple and rapid. Analyzing of actual samples and standard samples, the determination result of this method is consistent with that of standard methods (Flame atomic absorption spectroscopy). Thus the method had potential feasibility to analysis for Ag(I) in the environmental water samples, pharmaceutical, and food industries.

## Introduction

Due to the good antibacterial properties, silver ions and silver nanoparticles are widely used in medical treatment, food packaging, food storage containers and water treatment etc (1, 2). It is reported that the global production of silver nanoparticles is up to 600 tons per year (3). The widespread use of these products increases the chances of their release into the water environment and human exposure. Silver ions and nanoparticles can enter human body thought the food chain (4, 5). Huang et al. took a kind of commercial food fresh container (polyethylene plastic bags) as the research object and research silver nanoparticles in four different types of food simulation liquids (water, acid, alcohol, and oil), and they found that different degrees of migration occurred in all of them (6). Silver ions can enter the blood circulation and accumulate in other tissues and organs of the whole body. When reaching a certain value, they will produce toxic reactions such as hepatotoxicity, nephrotoxicity and neurotoxicity to the human body (7). It also may inhibit cell proliferation, produce cytotoxicity during use, and induce different types of cytopathic changes (8–11). Therefore, the development of a simple, rapid, efficient, and selective method for the detection of silver ions is of great significance to the food detection, the protection of the environment and human health, especially in the area of rapid food testing.

Erythrosine is a halogenated derivative of fluorescein, which is obtained by introducing four iodine atoms into the fluorescein. Due to the planarity and rigidity of molecular geometry and the large conjugated system, the parent molecules fluorescein have excellent fluorescence characteristics and high molar absorption coefficient. The analytical chemical properties of Ery was further improved by introducing I chromophores. Therefore, Ery have been widely used in absorption spectrum and fluorescence spectrum analysis, as well as in photon and electrochemistry. However, for a long time, Ery only have been applied to a kind of ionic association reagent, and cannot be directly used in the determination of metal ions. The metal ions must first form a larger chelate cation with a ligand, and then further form an ion-association with the Ery, which can be used to determine some material by absorption, fluorescence and resonance Rayleigh scattering spectroscopy (12–15). For example, Yi et al. reported that Pd(II) with Lincomycin formed a binary chelate and then bound with Ery to form a ternary ion-association for detecting Lincomycin (16). Tian et al. studied the interaction of erythrosine-phen-Cd(II) systems for testing Cd(II) (17). All of these methods are including a ternary complicated system and can’t directly be used to determine a certain substance.

In this study, we found that Ery could react with Ag(I) to form hydrophobic ion-association complex in pH 4.4∼4.6 weakly acidic media, which could further aggregate to form nanoparticles with an average particle size of about 45 nm under the action of water phase extrusion and van der Waals force. As a result, it could lead to a decrease of absorbance, a significant enhancement of resonance Rayleigh scattering (RRS) and the appearance of a new emission spectrum. Based on these phenomena, two new methods (spectrophotometry and RRS) were established for the determination of trace silver ion, by directly using Ery. The detection limits for silver ion by spectrophotometry and RRS were 9.74 and 0.12 ng/ml, respectively. The formation of nanoparticles, the optimum reaction conditions, the influence factors, the reason for enhancement of the scattering intensity and the effect of coexisting substance are investigated in this paper. This research showed that RRS method not only had good selectivity and high sensitivity, but also was simple and rapid. The detecting result is consistent with this of standard methods (Flame atomic absorption spectroscopy method), and this method could be used to the determination of Ag^+^ in actual samples and standard samples. Therefore, the method had potential feasibility to analysis for Ag(I) in the environmental water samples, pharmaceutical, and food industries. It can provide a new and rapid method for food safety testing.

## Experimental Sections

### Instrument and Reagents

The absorption spectra were recorded through a UV-8500 spectrophotometer (Shanghai Tianmei, China). The RRS spectra were obtained from the F-2500 fluorescence spectrophotometer (Hitachi, Tokyo, Japan). The morphologies and microstructures of the ion-association complex were gained using the transmission electron microscopy (FEI Company, Hillsboro, OR, United States).

A stock solution of erythrosin (Ery) (1.0 × 10^–3^mol/l, E. Merck.), Ag (I) (100 μg/ml, Institute of Standard Samples, Ministry of Environmental Protection, China) were prepared and kept at 4°C, respectively. Working solutions were freshly prepared by diluting the corresponding stock solutions.

### Procedure

1.0 ml Britton-Robinson buffer solution (BR), 1.0 ml of 2.5 × 10^–4^ mol/l Ery solution and a certain amount of Ag (I) were added into a 10 ml marked test tube. Then, it was fixed to the scale line with distilled water. The RRS intensity (*I*) and absorbance (*A*) were recorded at 324 and 552 nm respectively, and Δ*I* and Δ*A* were calculated.

## Results and Discussion

### Absorption Spectrum

**FIGURE 1:**
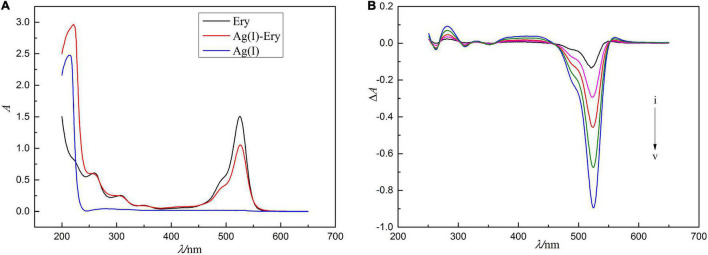
Concentration of erythrosine is 1.0 × 10^–5^ mol/l, pH = 4.4. In panel **(A)**, the absorption spectrum was recorded by using water as a reference solution, the absorption spectra were measured using the reagent blank as the reference solution in panel **(B)**, and the concentrations of Ag(I) from curve i to v are 0.500, 0.625, 0.750, 0.875, and 1.00 μg/ml.

The absorption spectra of Ag(I), Ery and their combined products are shown in Figure 1. As shown in Figure 1A, the maximum absorption wavelength of Ery is 524 nm, while Ag(I) itself has almost no light absorption in the range of 300–700 nm. When Ag(I) reacts with Ery to form the ion-association, the absorption spectrum changes (see Figure 1B). The absorbance at 522 nm decreases significantly, and the violet shift is 2 nm compared with the maximum absorption peak of Ery, while two new absorption peaks appear at 280 and 560 nm, and the change of absorbance at 552 nm is in a linear relationship with Ag(I) concentration. The molar absorption coefficient (ε) is 1.2 × 10^5^ L/mol^/^cm, and the detection limit is 9.74 ng/ml. So, a new spectrophotometric method for the detection of Ag(I) can be established. Although absorption method has high sensitivity, the signal is to use subtractive signal, and so it is not ideal methodologically, a better method need be found.

### Resonance Rayleigh Scattering Spectrum

**FIGURE 2:**
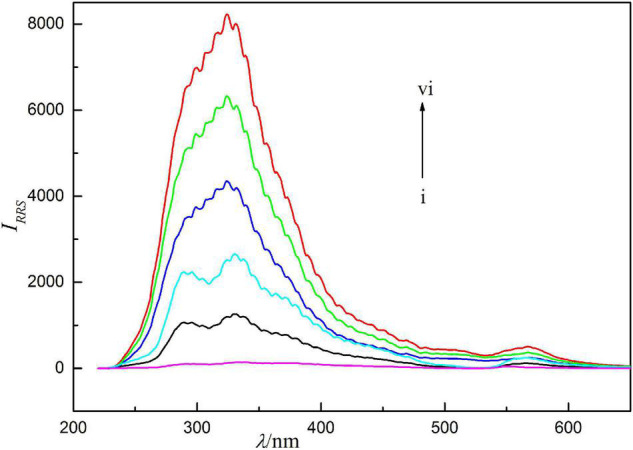
Resonance Rayleigh scattering (RRS) spectra, concentration of erythrosine is 2.5 × 10^–5^ mol/l, pH = 4.4, concentrations of Ag(I) from curve i to vi are 0, 0.125, 0.250, 0.375, 0.500, and 0.625 μg/ml.

Resonance Rayleigh scattering spectra of the reaction system are shown in Figure 2. It can be seen from the figure that the RRS signal of Ag(I) and Ery themselves are extremely weak. When they react to generate binding products, the RRS spectra are significantly enhanced, and a strong scattering spectral band appears near 286–375 nm, with the maximum scattering peak at 324 nm and a weak scattering peak at 566 nm. *I*_*RRS*_ at 324 nm is strengthened with the increase of Ag(I) concentration and show a linear relationship in the range of 0.0039–0.75 μg/ml. The detection limit of RRS method was 0.12 ng/ml. Compared with spectrophotometry, its sensitivity is higher. Therefore, it is more beneficial to measure silver ions. This method avoids the disadvantages of spectrophotometry.

### Optimization Reaction Conditions

#### Effect of pH

Three kinds of buffer solution (BR, HCl-sodium citrate, and HCl-NaAc) were used as reaction medium to test their effects on RRS, and the results showed that BR buffer solution was the best. When BR buffer solution was used, Δ*I*_*RRS*_ reached the maximum in the range of pH 4.4–4.6. And 1.0 ml BR buffer solution was the best dosage. When the pH is 4.4–4.6, the hydroxy of Ery will dissociate, Ag(I) combines with it at this position to form ion-association, further aggregates in the solution to form nanoparticles. Beyond this range, the dissociation position of Ery is different, and the corresponding ion-association cannot be formed without phenomenon.

#### Effect of Erythrosine Concentration

The results showed that Δ*I*_*RRS*_ reached the biggest when the concentration of Ery was 2.5 × 10^–5^ mol/l, and it would decrease when the concentration was too low or too high. Because the concentration of Ery was too low, the reaction was incompleted. However, if the concentration of Ery was large, the aggregation of the dye itself resulted in the decrease of Δ*I*_*RRS*_.

#### Reaction Speed and Stability

At room temperature, the reaction of the system has become stable in 5 min and the scattering intensity can be keep for about 12 h. Therefore, the system has good stability.

### Reaction Mechanism of Ag(I) and Erythrosine

#### Ion-Association Reaction

The hydroxy of Ery dissociates in the pH 4.4 solution, Ag(I) combines with it at this position to form ion-association with five-membered ring structure. The composition ratio of Ery to Ag(I) was determined by equimolar continuous change method and the molar ratio method, respectively. The results showed that Ery and Ag(I) formed a 1:1 electroneutral ion-association.

Although, Ery is a binary weak acid (H_2_L), the calculation results show that Ery exists mainly in the form of monovalent anion (HL^–^) according to pK_a 1_ = 3.6, in pH 4.4 medium. Theoretically, HL^–^ can be obtained by the dissociation of hydroxyl or carboxyl groups on the benzene ring.

If there is no strong electron-withdrawing groups, the carboxyl will dissociate before hydroxy (18, 19). But the opposite happens. After two strong electron-absorbing groups (iodine atom) are introduced into the o-position of the hydroxyl on the xanthene ring, they have an electron-absorbing effect on the hydroxyl, reduce its negative charge density and make it easier to dissociate from the hydroxyl oxygen atoms than the carboxyl groups on the phenyl. So, the hydroxy of Ery will dissociate.

**FIGURE 3:**
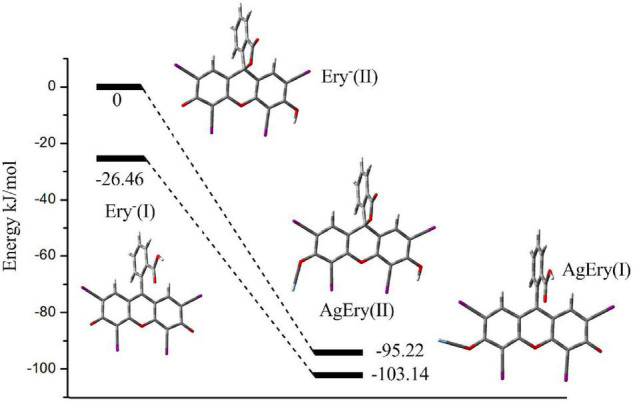
The reaction coordination and energy surface of Ag(I) and Ery^–^ system.

To further confirming the correctness of hydroxyl dissociation, we calculated the reaction system by the density functional method of quantum chemistry (B3LYP). We used LanL2DZ pseudopotential basis for I atom and Ag atom, and 6–31g (d) basis for other atoms. Considering the effect of the solvent on the system, we adopted the polarimetric continuum solvation model (PCM) in aqueous solution to optimize the whole calculation process. When H^+^ dissociate from Ery, its anions (Ery^–^) is formed, which may have two constructions. One possibility is that the dissociation of the H atom located on the carboxyl group on the benzene ring, produce benzoic acid [Ery^–^ (I)] (see Figure 3). The other one is that when the H atom located on the carbonyl group next to I atom dissociate from Ery, the oxygen on the carboxyl group of the benzene ring combines with the central carbon atom on the parallel ring to form a five-membered ring structure [Ery^–^ (II)] (see Figure 3). Through full optimization calculation, it is found that the energy of the first structure is 26.46 kJ/mol lower than that of the second structure, indicating that the first structure is more stable than the second structure and is the main form of Ery^–^ in solution.

**FIGURE 4:**
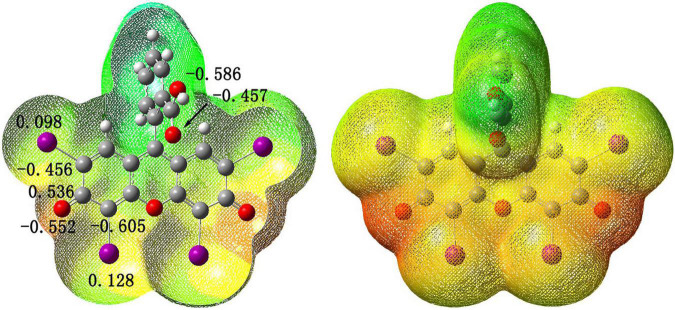
Potential electronic diagram and selected atomic charges of Ery^–^.

At the same time, in order to further understand the interaction position of Ag^+^ and Ery^–^, we also calculated the electrostatic potential diagram of Ery^–^. The electrostatic potential diagram and part of the atomic charge of Ery^–^ are showed in Figure 4. The results showed that the negative charge of Ery^–^ was mainly concentrated in the carbonyl position between iodine atoms, so Ag^+^ should combine with it at this position to form ion-association. The energy change of binding process and the final ion-association were also calculated (see Figure 3). Since Ery^–^ had two structures, we calculated the energy changes of the two structures. The energy of type [Ag-Ery (I)] was 7.92 kJ/mol lower than that of type [Ag-Ery (II)]. Therefore, whether it existed alone or formed an ion-association, the energy of type [Ag-Ery (I)] was lower than that of type [Ag-Ery (II)], so the H atom on the carbonyl group next to the iodine atom should ionize.

**FIGURE 5:**
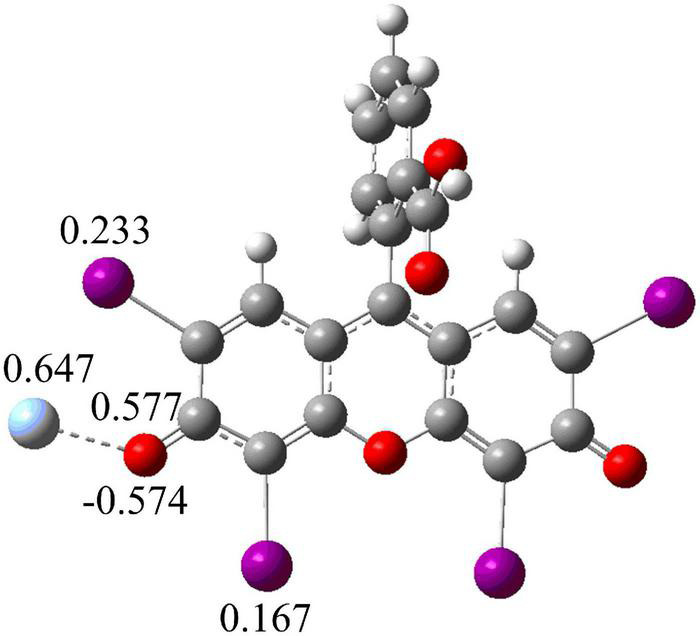
Structure and selected atomic charges of Ag-Ery.

When Ag^+^ directly acted on the oxygen atom in the large conjugate system, due to the induction effect, the charge of iodide ions changed greatly from 0.098 and 0.128 to 0.233 and 0.167 (see Figure 5), respectively, which was resulted in the change of absorption spectrum (see Figure 1A).

#### The Formation of Nanoparticles

We also calculated the volume and surface area of Ery and the bond length of the ion-association by using B3LYP. The molar volume was 342.403 cm^3^/mol, and the molecular surface area was 498.047 Å^2^/molecule. When the molecule was calculated as a sphere, its diameter should be 1.25 nm according to its area. The bond length of Ag-Ery was 0.228 nm, and the radius of Ag^+^ was 0.126 nm, so the diameter of ion-association should be less than 2 nm.

**FIGURE 6:**
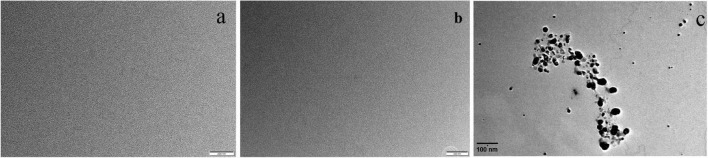
Transmission electron micrographs for **(a)** Ag(I), **(b)** Ery^–^, and **(c)** the products of the reaction for Ery and Ag(I).

However, transmission electron microscopy (TEM) was used to observe the surface texture of Ery, Ag^+^ and ion-association, respectively, and the results are shown in the Figure 6. There were not any nanoparticles of Ery (Figure 6a) and Ag^+^ (Figure 6b), which corresponded to the calculated results. However, when the ion-association was formed, some substances with average particle size of 45 nm were observed (Figure 6c). That is to say, Ag-Ery ion-association do not exist as a single molecule, but further aggregated in the solution to form nanoparticles. So, the end products of the reaction for Ery and Ag^+^ are some nanoparticles.

From what has been discussed above, the mechanism of reaction is that the hydroxy of Ery will dissociate firstly, then Ag(I) combines with it at this position to form ion-association with five-membered ring structure, finally further aggregated in the solution to form nanoparticles.

#### Reasons for Resonance Rayleigh Scattering Enhancement

##### Influence of Absorption Spectrum on Resonance Rayleigh Scattering

**FIGURE 7:**
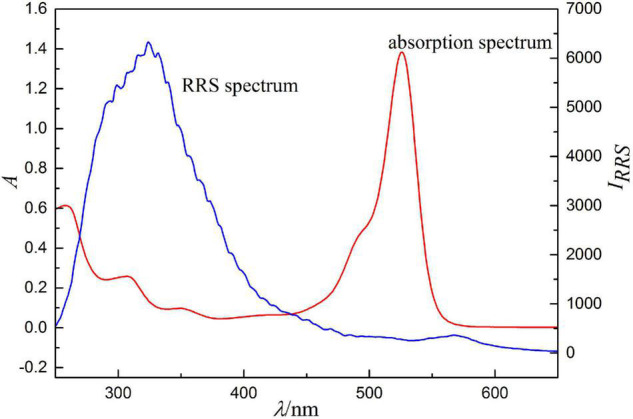
Absorption spectra and RRS spectra, concentration of erythrosine is 2.5 × 10^–5^ mol/l, pH = 4.4, concentrations of Ag(I) is 0.500 μg/ml.

Because RRS is a scattering-absorption-rescattering process generated by resonance between scattering and light absorption, RRS spectrum should be closely related to absorption spectrum, which is a necessary condition for the generation of RRS. The comparison between RRS and absorption spectrum (Figure 7) shows that RRS is located in its absorption band. The RRS peaks of the ion association near 324 and 566 nm have a good correspondence with the absorption peaks near 280 and 526 nm, respectively. Therefore, resonance enhancement effect is generated and the scattering intensity increases significantly.

##### Formation of Hydrophobic Interface

Whether Ery or Ag (I), they have the strong hydrophilicity and can well dissolve in water. When Ery and Ag (I) react to form ion-association and further aggregate to form nanoparticles, there is a liquid-solid interface between these products and water phase, which will lead to a surface-enhanced scattering effect and significantly enhance the scattering.

##### The Molecular Volume Increases

It is known that the increase of molecular volume is conducive to the improvement of scattering intensity. When nanoparticles are formed, their average particle size is 45 nm, and the molecular volume increases dramatically, which is also helpful to the enhancement of scattering.

### Standard Curve

Under the optimum experimental conditions, the Δ*I*_*RRS*_ and Δ*A* value with concentration of Ag (I) were investigated. All the parameters of the standard curves and the limits of detection (DL) are listed in Table 1. The results show that the detection limit of RRS method is 0.12 ng/ml, that of spectrophotometry is 9.74 ng/ml. Therefore, RRS method is more sensitive than spectrophotometry. Compared with the common colorimetry, fluorescence, atomic absorption spectrometry, flame atomic absorption spectroscopy and electrochemical methods, the detection limit of this method is the lowest with the number range from many times to thousands times (see Table 2). In addition, the method is simple, rapid and low cost, which is more beneficial to the determination of silver ions.

**TABLE 1:** Related parameters of the calibration graphs and the detection limits.

Method	Mesurement wavelength	Linear regression equation (μg/mL)	Correlation coefficient (r)	Linear range (μg/mL)	Detection limits 3σ (ng/mL)
RRS	λ*ex/*λ*em* = 324/324 nm	Δ*I* = 1.32 × 10^4^*c*−458	0.9969	0.0039∼0.75	0.12
SP*	λ = 522 nm	Δ*A* = −1.54*c* + 0.67	0.9994	0.032∼1.0	9.74

SP* is spectrophotometry.

**TABLE 2:** Comparison of different methods for Ag^+^ detection.

Methods	Detection limits (ng/mL)	References
Atomic absorption and plasma emission spectrometry	473	1 (20)
Atomic absorption spectrometry	1.1	2 (21)
Flame atomic absorption spectroscopy	4.6	3 (22)
Flame atomic absorption spectroscopy	30	4 (23)
Fluorescence	14	5 (24)
Fluorescence	43	6 (25)
Fluorescence	5	7 (26)
Fluorescence	200	8 (27)
Colorimetry	6.9	9 (28)
Colorimetry	182	10 (29)
Electrochemical methods	3.3	11 (30)
Electrochemical methods	2.1	12 (31)
RRS	6.3	13 (32)
RRS	21.6	14 (33)
Spectrophotometry	9.74	Present work
RRS	0.12	Present work
		

### Selectivity and Analytical Application

#### Selectivity of the Method

Under the optimal conditions, the influence of coexisting ions on the determination of Ag(I) was investigated, and the results were shown in Table 3. The concentration of common inorganic acid ions (such as SO_4_^2–^, NO_3_^–^, and PO_4_^3–^) can reach 400–500 times, that of alkali metal ions (Na^+^, K^+^) and NH_4_^+^ can reach 800–1,000 times, and that of metal ions, including Cu(II), Pb(II), Cd(II), Hg(II), etc., can reach more than 200 times. In other words, under the experimental conditions, Ery only reacts with monovalent silver ion, not other high-valence metal ions, so the method has good selectivity and could be used for the determination of trace Ag(I) in actual samples.

**TABLE 3:** Effects of coexisting substances (c = 0.50 μg/mL).

Coexisting substance	Times	Ralative error (%)	Coexisting substance	Times	Ralative error (%)	Coexisting substance	Times	Ralative error (%)
NO_3_^–^	500	2.5	Mg(II)	800	2.5	Fe(III)	500	2.4
Cl^–^	20	2.4	Pb(II)	700	0.6	Al(III)	400	−4.3
SO_4_^2–^	400	3.1	Cd(II)	600	3.8	Au(III)	200	3.2
PO_4_^3–^	500	3.5	Zn(II)	900	−2.6	Sb(III)	300	1.9
NH_4_^+^	1000	−4.2	Mn(II)	500	2.4	Bi(III)	300	1.2
Na^+^	800	2.6	Hg(II)	200	1.8	Ir(III)	200	2.5
K^+^	800	−3.4	Ni(II)	400	4.0	Rh(III)	400	3.4
Pd(II)	400	2.1	Co(II)	400	2.9	Pt(IV)	500	2.7
Ca(II)	500	−3.0	Cu(II)	700	1.2	W(VI)	400	1.9

#### Analysis of Actual Samples

Resonance Rayleigh scattering method was used for the detecting Ag(I) in mineral drinks and environment water samples, and the results were shown in Table 4. The environmental water samples were obtained from two drinking water source sections of Jialing River and two sections of Longfengxi, a tributary of Jialing River. For the detecting of mineral drinks samples, the relative standard deviations were 1.43 ∼ 3.46%, and the average recoveries were 93.3 ∼ 140.0%. For the determination of environment water samples, the relative standard deviations were 1.53 ∼ 2.51%, and the average recoveries were 95.0 ∼ 103.3%. The results were in accord with those of the standard method (FAAS). These results revealed that the RRS method could be applied to the analysis of real samples and environment water samples to ensure food safety.

**TABLE 4:** Results for the determination of Ag(I) in mineral drinks and environment water samples.

Sample	Found amount (μg/mL)	FAAS^#^ method (μg/mL)	Added amount (μg/mL)	Found total amount (μg/mL)	RSD (%)	Recovery (%)
Mineral drink 1	ND*	ND*	0.20	0.21	1.43	105.0
Mineral drink 2	ND*	ND*	0.30	0.28	2.67	93.3
Mineral drink 3	ND*	ND*	0.40	0.40	3.09	100.0
Mineral drink 4	ND*	ND*	0.50	0.53	3.28	106.0
Jialing river Beiwenquan Section	ND*	ND*	0.20	0.20	2.02	100.0
Jialing river Shuitu Section	ND*	ND*	0.30	0.31	1.53	103.3
Liangtan river Longfeng Section	ND*	ND*	0.40	0.38	2.33	95.0
Liangtan river Xixiqiao Section	ND*	ND*	0.50	0.49	2.51	98.0

ND* is not detected. FAAS^#^ is Flame atomic absorption spectroscopy. n = 5.

#### Analysis of Standard Samples

The method was validated with the standard samples (Institute for Environmental Reference Materials of Ministry of Environmental Protection), and the results were listed in Table 5. The relative errors were 2.00 ∼ 5.00%, and there was no significant difference with standard values (α = 0.05). Therefore, the method has good accuracy and repeatability for the determination of Ag(I) in the standard samples.

**TABLE 5:** Results for the determination of Ag(I) in Certified Reference Material.

Sample number	Found amount (μg/mL)	FAAS^#^ method (μg/mL)	Standard values (μg/mL)	Relative error (%)
204206	0.440	0.441	0.449	2.00
GSBZ50038-95	0.190	0.192	0.200	5.00

*FAAS^#^ is Flame atomic absorption spectroscopy. n = 5.*

## Conclusion

Erythrosine can react with Ag(I) to form hydrophobic ion-association complex, which can further aggregate to form nanoparticles. The reaction can lead to a decrease of absorbance and a significant enhancement of resonance Rayleigh scattering (RRS) Based on these, spectrophotometry and RRS method were developed for the detection of Ag(I). When we used RRS mothod to determine silver ions in mineral water samples, environment water samples and standard samples, the results were consistent with the standard method. The analysis for actual samples can be done in several minutes, and RRS has a potent ability to be used for the rapid detection of trace Ag(I) in other food samples. In addition, without the need for the other reagents, RRS can be used to directly detecting Ag(I), and the process of determination is simple and environmentally friendly.

## Data Availability Statement

The original contributions presented in the study are included in the article/Supplementary Material, further inquiries can be directed to the corresponding authors.

## Author Contributions

JW: methodology, experiment, data collecting, and writing—origin draft preparation. SL: supervision. WS: quantum computation and writing—reviewing and editing. All authors contributed to the article and approved the submitted version.

## Conflict of Interest

The authors declare that the research was conducted in the absence of any commercial or financial relationships that could be construed as a potential conflict of interest.

## Publisher’s Note

All claims expressed in this article are solely those of the authors and do not necessarily represent those of their affiliated organizations, or those of the publisher, the editors and the reviewers. Any product that may be evaluated in this article, or claim that may be made by its manufacturer, is not guaranteed or endorsed by the publisher.

## References

[1] Sharma VK Siskova KM Zboril R Gardea-Torresdey JL. Organic-coated silver nanoparticles in biological and environmental conditions: fate, stability and toxicity. *Adv Colloid Interface Sci.* (2014) 204:15–34. 10.1016/j.cis.2013.12.002 24406050

[2] Zhang C Hu Z Deng B. Silver nanoparticles in aquatic environments: physiochemical behavior and antimicrobial mechanisms. *Water Res.* (2016) 88:403–27. 10.1016/j.watres.2015.10.025 26519626

[3] Temizel-Sekeryan S Hicks AL. Global environmental impacts of silver nanoparticle production methods supported by life cycle assessment. *Resour Conserv Recycl.* (2020) 156:104676. 10.1016/j.scitotenv.2015.02.042 25728395

[4] McTeer J Dean AP White KN Pittman JK. Bioaccumulation of silver nanoparticles into Daphnia magna from a freshwater algal diet and the impact of phosphate availability. *Nanotoxicology.* (2014) 8:305–16. 10.3109/17435390.2013.778346 23421707

[5] Lekamge S Miranda AF Ball AS Shukla R Nugegoda D. The toxicity of coated silver nanoparticles to Daphnia carinata and trophic transfer from alga Raphidocelis subcapitata. *PLoS One.* (2019) 14:e0214398. 10.1371/journal.pone.0214398 30943225 PMC6447189

[6] Huang Y Chen S Bing X Gao C Wang T Yuan B. Nanosilver Migrated into Food-Simulating Solutions from Commercially Available Food Fresh Containers. *Packag Technol Sci.* (2011) 24:291–7. 10.1002/pts.938

[7] Alt V Bechert T Steinrucke P Wagener M Seidel P Dingeldein E An in vitro assessment of the antibacterial properties and cytotoxicity of nanoparticulate silver bone cement. *Biomaterials.* (2004) 25:4383–91. 10.1016/j.biomaterials.2003.10.078 15046929

[8] Burd A Kwok CH Hung SC Chan HS Gu H Lam WK A comparative study of the cytotoxicity of silver-based dressings in monolayer cell, tissue explant, and animal models. *Wound Repair Regen.* (2007) 15:94–104. 10.1111/j.1524-475X.2006.00190.x 17244325

[9] Yates CC Whaley D Babu R Zhang J Krishna P Beckman E The effect of multifunctional polymer-based gels on wound healing in full thickness bacteria-contaminated mouse skin wound models. *Biomaterials.* (2007) 28:3977–86. 10.1016/j.biomaterials.2007.05.008 17561250 PMC2034502

[10] Atiyeh BS Costagliola M Hayek SN Dibo SA. Effect of silver on burn wound infection control and healing: review of the literature. *Burns.* (2007) 33:139–48. 10.1016/j.burns.2006.06.010 17137719

[11] Gong H Li X. Y-type, C-rich DNA probe for electrochemical detection of silver ion and cysteine. *Analyst.* (2011) 136:2242–6. 10.1039/c1an15159b 21512696

[12] Abdel-Lateef MA Omar MA Ali R Derayea SM. Xanthene based spectroscopic probe for the analysis of HCV antiviral, daclatasvir dihydrochloride, through feasible complexation reaction. *Microchem J.* (2019) 145:672–5. 10.1016/j.microc.2018.11.038

[13] Wang J Liu Z Liu J Liu S Shen W. Study on the interaction between fluoroquinolones and erythrosine by absorption, fluorescence and resonance Rayleigh scattering spectra and their application. *Spectrochim Acta A Mol Biomol Spectrosc.* (2008) 69:956–63. 10.1016/j.saa.2007.05.057 17618827

[14] Liu JF Li NB Luo HQ. Resonance Rayleigh scattering, second-order scattering and frequency doubling scattering spectra for studying the interaction of erythrosine with and its analytical application. *Spectrochim Acta Part A Mol Biomol Spectrosc.* (2011) 79:631–7. 10.1016/j.saa.2011.03.046 21536488

[15] Hamad AA Ali R Ali HRH Dalia M Derayea SM. Facile complexation reactions for the selective spectrofluorimetric determination of albendazole in oral dosage forms and spiked human plasma. *RSC Adv.* (2018) 8:5373–81. 10.1039/c7ra12360d 35542411 PMC9078121

[16] Yi A Liu Z Liu S Hu X. Study on the interaction between palladium(II)-lincomycin chelate and erythrosine by absorption, fluorescence and resonance Rayleigh scattering spectra and its analytical applications. *Luminescence.* (2009) 24:23–9. 10.1002/bio.1057 18785616

[17] Tian J Zhang Q Liu S Yang J Teng P Zhu J Study on erythrosine-phen-Cd(II) systems by resonance Rayleigh scattering, absorption spectra and their analytical applications. *Spectrochim Acta A Mol Biomol Spectrosc.* (2015) 140:15–20. 10.1016/j.saa.2014.12.003 25579798

[18] Matveets MA Akhmetova SD. Investigation of the spectrophotometric and luminescence properties of hydroxyxanthene dyes in aqueous-solutions. *J Anal Chem Ussr.* (1979) 34:807–12.

[19] McHedlov-Petrossyan NO Mayorga RS. Extraordinary character of the solvent influence on protolytic equilibria: inversion of the fluorescein ionization constants in H2O–DMSO mixtures. *J Chem Soc Faraday Trans.* (1992) 88:3025–32. 10.1039/ft9928803025

[20] Rains TC Watters RL Epstein MS. Application of atomic absorption and plasma emission spectrometry for environmental analysis. *Environ Int.* (1984) 10:163–8. 10.1016/0160-4120(84)90092-8 33395953

[21] Mohammadi SZ Afzali D Heshmati Z. Ligand-lessin situsurfactant-based solid phase extraction for preconcentration of silver from natural water samples prior to its determination by atomic absorption spectroscopy. *Toxicol Environ Chem.* (2014) 95:1299–308. 10.1016/j.jhazmat.2008.09.050 18977595

[22] Soylak M Cay RS. Separation/preconcentration of silver(I) and lead(II) in environmental samples on cellulose nitrate membrane filter prior to their flame atomic absorption spectrometric determinations. *J Hazard Mater.* (2007) 146:142–7. 10.1016/j.jhazmat.2006.12.005 17196741

[23] Mondal BC Das D Das AK. Application of a new resin functionalised with 6-mercaptopurine for mercury and silver determination in environmental samples by atomic absorption spectrometry. *Anal Chim Acta.* (2001) 450:223–30. 10.1016/s0003-2670(01)01385-x

[24] El-Shekheby HA Mangood AH Hamza SM Al-Kady AS Ebeid el ZM. A highly efficient and selective turn-on fluorescent sensor for Hg2+, Ag+ and Ag nanoparticles based on a coumarin dithioate derivative. *Luminescence.* (2014) 29:158–67. 10.1002/bio.2521 23703858

[25] Shen L Chen M Hu L Chen X Wang J. Growth and stabilization of silver nanoparticles on carbon dots and sensing application. *Langmuir.* (2013) 29:16135–40. 10.1021/la404270w 24308456

[26] Qu F Liu J Yan H Peng L Li H. Synthesis of organic nanoparticles of naphthalene–thiourea–thiadiazole-linked molecule as highly selective fluorescent and colorimetric sensor for Ag(I). *Tetrahedron Lett.* (2008) 49:7438–41. 10.1016/j.tetlet.2008.10.077

[27] Freeman R Finder T Willner I. Multiplexed analysis of Hg2+ and Ag+ ions by nucleic acid functionalized CdSe/ZnS quantum dots and their use for logic gate operations. *Angew Chem Int Ed Engl.* (2009) 48:7818–21. 10.1002/anie.200902395 19746491

[28] Zhou XH Kong DM Shen HX. Ag+ and cysteine quantitation based on G-quadruplex-hemin DNAzymes disruption by Ag+. *Anal Chem.* (2010) 82:789–93. 10.1021/ac902421u 20039758

[29] Liu L Lin H. Paper-based colorimetric array test strip for selective and semiquantitative multi-ion analysis: simultaneous detection of Hg(2)(+), Ag(+), and Cu(2)(+). *Anal Chem.* (2014) 86:8829–34. 10.1021/ac5021886 25070403

[30] Liu Q Li J Yang W Zhang X Zhang C Labbe C Simultaneous detection of trace Ag(I) and Cu(II) ions using homoepitaxially grown GaN micropillar electrode. *Anal Chim Acta.* (2020) 1100:22–30. 10.1016/j.aca.2019.11.010 31987144

[31] Romih T Hočevar SB Jemec A Drobne D. Bismuth film electrode for anodic stripping voltammetric measurement of silver nanoparticle dissolution. *Electrochim Acta.* (2016) 188:393–7. 10.1016/j.electacta.2015.11.110

[32] Wen G Lin C Tang M Liu G Liang A Jiang Z. A highly sensitive aptamer method for Ag+sensing using resonance Rayleigh scattering as the detection technique and a modified nanogold probe. *RSC Adv.* (2013) 3:1941–6. 10.1039/c2ra22542e

[33] Zhan S Wu Y He L Wang F Zhan X Zhou P A silver-specific DNA-based bio-assay for Ag(i) detection via the aggregation of unmodified gold nanoparticles in aqueous solution coupled with resonance Rayleigh scattering. *Anal Methods.* (2012) 4:3997. 10.1039/c2ay25403d

