# Supplementary material for: Absorption and Resonance Rayleigh Scattering Spectra of Ag(I) and Erythrosin System and Their Analytical Application in Food Safety
Source: Front Nutr. 2022 May 9;9:900215. doi: 10.3389/fnut.2022.900215 (PMC9125220; doi:10.3389/fnut.2022.900215)
Supplement: Supplementary file 1 [file Data_Sheet_1.docx]

Supplementary Material

**Absorption and Resonance Rayleigh Scattering Spectra of** **Ag(I) and** **Erythrosin System and Their Analytical Application In Food Safety**

Jian Wang, Shaopu Liu, Wei Shen


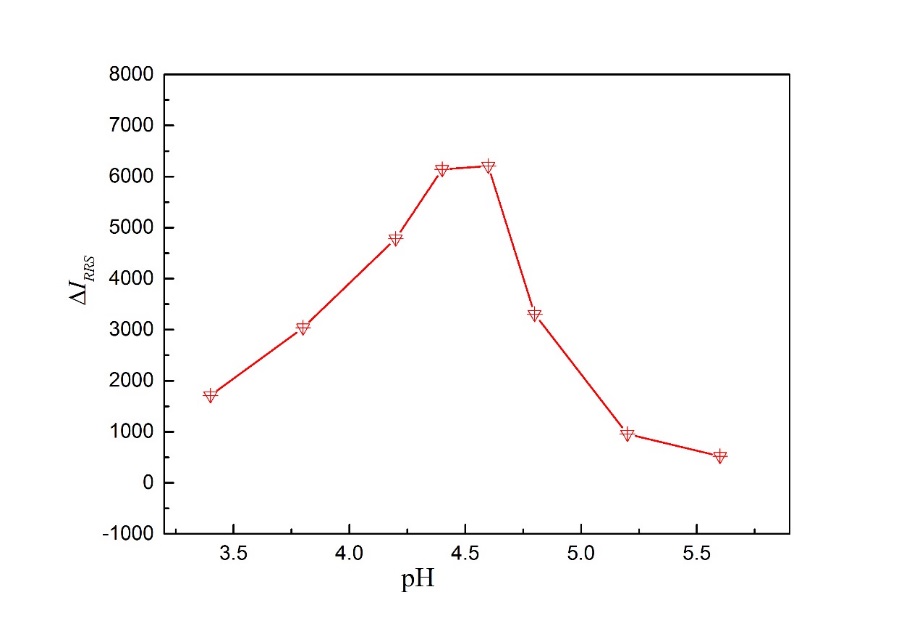


**Fig. 1s** Effect of pH on RRS spectra, concentration of erythrosine is 2.5×10^-5^ mol/l, pH=4.4, concentrations of Ag(I) is 0.500 µg/ml.


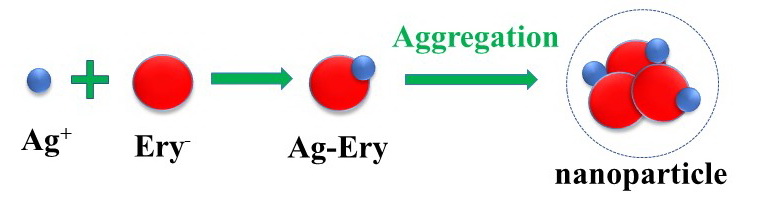


**Fig. 2s** The scheme of reaction mechanism
